# Child Attachment Representations and Parenting Stress in Mothers and Fathers of School-Age Children with a Diagnosis of Autism Spectrum Disorder: A Pilot Cross-Sectional Study

**DOI:** 10.3390/children10101633

**Published:** 2023-09-30

**Authors:** Michele Giannotti, Paola Venuti, Simona De Falco

**Affiliations:** Department of Psychology and Cognitive Science, Observation, Diagnosis and Education Lab, University of Trento, Via Matteo del Ben 5/b, 38068 Rovereto, TN, Italy; michele.giannotti@unitn.it (M.G.);

**Keywords:** attachment, autism spectrum disorder, parenting stress, ASD severity, mothers, fathers

## Abstract

Mothers and fathers of autistic children (ASD) tend to report elevated levels of parenting stress. Thus, it is critically important to understand which factors contribute to an imbalance between the perceived demands of parenting and the available psychological resources. To date, little is known about the association between child attachment representations and parenting stress. In this study, we first examined group differences in parenting stress levels based on child diagnosis and parents’ gender. Second, we explored the predictive role of child diagnosis, autism severity, and child attachment representations on parenting stress. The study involved 23 school-age children with ASD (IQ > 70), 27 without ASD (7–13 years), and their mothers (*n* = 50) and fathers (*n* = 50). Data were collected from 2017 to 2020. Parents completed the Social Responsiveness Scale 2 and the Parenting Stress Index—Short Form, while the children’s attachment representations were assessed using the School-age Assessment of Attachment. Parents of children with ASD reported higher stress compared with controls. No differences were found between mothers and fathers. Implicit attachment representations have been found to be associated with parenting stress only in mothers, while the severity of social impairment showed a significant effect on parenting stress in both parents. These findings revealed the potential benefit of adaptive attachment representations not only for children themselves but also for mothers and the family system, suggesting the bidirectional nature of parent–child relationships in the context of ASD. The uniqueness of maternal and paternal parenting experiences should be considered when parenting stress is addressed.

## 1. Introduction

Parenting stress encompasses a variety of negative feelings and adverse responses arising from the difficulties of balancing personal psychological resources and parenting demands [1]. Previous research on parenting stress has often focused on families of children with developmental disabilities, particularly autism spectrum disorder (ASD) [2], since rearing a child with a neurodevelopmental disorder may present additional difficulties for parents.

Specifically, ASD is characterized by early-onset and persistent impairments in social communication, as well as restricted and repetitive patterns in behaviors, interests, and activities [3]. Due to the pervasive and long-term impact of these characteristics on parenting and child developmental trajectory, mothers and fathers of children with ASD may face unique and multiple challenges [4,5]. Thus, examining the child factors that contribute to the experience of parental stress may provide useful insights for tailoring specific family interventions aimed at supporting parents of children with ASD.

### 1.1. Parental Stress in Mothers and Fathers of Children with ASD

Communicative difficulties and impairments in social reciprocity and interpersonal relatedness, including restrictive interests and repetitive behaviors that characterize ASD diagnosis, may increase parental burden and demands, regardless of the level of cognitive functioning [6]. Prior research established that parents of children with ASD reported a higher level of stress compared with those of children with typical development (TD) [7] and other developmental disabilities or special health care needs [8]. Focusing on parents’ gender, several studies documented similar levels of parenting stress in mothers and fathers of children with ASD [5,9,10], despite some contradictory findings. For instance, a study by Rivard and colleagues [11] showed that fathers reported higher perceived parenting stress than mothers measured via the Parenting Stress Index—Short Form (PSI/SF; Abidin 1995). By contrast, Dabrowska and Pisula [12], using the Questionnaire of Resources and Stress (QRS) [13], found that mothers scored higher than fathers on 2 dimensions out of 11 (dependency and management; limits on family opportunities).

However, only a small number of studies focused on fathers including balanced samples [14]; thus, further investigations are needed to clarify potential gender differences in parenting stress.

Furthermore, prior research examined the role of specific predictors associated with parenting stress in the context of ASD, distinguishing between parental, child, and environmental factors [15]. Most of the literature on this topic drew attention to the influence of child characteristics on the experience of parenting stress, focusing mainly on mothers. Due to the heterogeneity of ASD, the predictive effect of child cognitive functioning and severity of ASD symptoms on parenting stress has been widely investigated. Firstly, parents of children with ASD appear to be at increased risk for parenting stress independently from the presence of intellectual disability [8,16]. On the other hand, most of the studies pointed out that higher severity of autism is associated with greater parenting stress [9,17]. Interestingly, Rivard and colleagues [11] found that ASD symptom severity predicted parental stress in fathers but not in mothers. On this matter, Davis and Carter [9] reported that child factors may have a different impact on mothers and fathers, suggesting the need to investigate their specific predictive effect separately for both parents.

In addition, further studies showed the contribution of other child dimensions that account for variations in parenting stress, such as executive functions [18] and behavioral difficulties [19,20]. Nevertheless, focusing on other child factors that are not related to ASD core symptoms, very little is known about the association between child attachment and parenting stress, particularly during middle childhood [21]. Attachment in young children has been defined as a disposition to seek and maintain proximity and contact with a selective adult figure in case of fear and threats or illness [22]. Beyond infancy, parents remain a consistent source of security for the child throughout childhood. The child’s internal representation of their past interactions with caregivers might shape their understanding of social situations and their expectations concerning relationships as well as dangerous circumstances [23].

### 1.2. Child Attachment in Cognitively Able School-Age Children with ASD and Parental Stress

Since attachment and social motivations share common underlying biobehavioral mechanisms [24], there was a debate about the coexistence of ASD symptoms and the development of secure attachment. Children with insecure attachment exhibit a wide range of behaviors that may also occur in ASD such as rigid and atypical play, inflexibility or difficulties in reflective functioning, emotion regulation, and sensory integration [25].

Early metanalytic findings [26] revealed that preschool children with ASD showed less secure attachment than comparison children. Nevertheless, this difference was observed only in children with intellectual disability. Consistently, previous studies on school-age children found that cognitively able children with a diagnosis of ASD revealed no differences in attachment security compared with the non-ASD group [24,25]. However, the few studies available on middle childhood were only based on self-reported questionnaires assessing perceived attachment to parents as reported by the child [27,28].

In this regard, it is essential to note that the assessment of attachment during this developmental stage is particularly puzzling, since the attachment system evolves as children develop, reflecting the impact of profound changes that occur at physical, psychological, and interpersonal levels [29,30]. Older children are less focused on physical proximity, while they are more able to engage in a cooperative partnership depending on parent accessibility. Moreover, they show an increased ability to regulate their states relying upon their internal attachment representations (originally defined as the Internal Working Model), which becomes more elaborated and internalized [31]. For this reason, different approaches and methods have been proposed for assessment, including observational measures, projective/semi-structured interviews, and secure base scripts [32].

Although it has been assumed that attachment during middle childhood plays a key role in children’s adjustment, little attention has been paid to the contribution of internal representations in the context of ASD [33]. More specifically, Goodman and Glenwick [34] found that affective attachment, but not parental perceptions of their child’s attachment to them, contributed negatively to parenting stress in both mothers and fathers. However, attachment has been assessed only by using parent-reported measures, providing a limited understanding of the construct and its relation to parenting stress.

To only study available considering the child perspective [21] found no significant associations between the perception of child attachment security and parenting stress, and no associations were found between child attachment and parenting stress in either the TD or the ASD groups. In this regard, the accuracy of self-reported measures of attachment has been questioned since they are not sensitive enough to capture children’s internal representations, which are mainly automated and unconscious [33]. Therefore, it has been suggested to adopt a multi-modal assessment of child attachment by integrating self-report and semi-structured interviews [31]. Moreover, prior research has focused on maternal stress, thus the influence of child attachment on paternal-reported stress in the context of ASD is yet to be explored.

### 1.3. The Current Study

The present case-control study sought to examine the relations between child attachment representations, assessed using a semi-structured interview, and parenting stress in mothers and fathers of school-age children with ASD (without intellectual disability). The first aim of the study was to examine differences in the level of parenting stress between groups, based on child diagnosis and parents’ gender. We hypothesized that parents of children with ASD showed higher levels of stress compared with those of TD children in line with the previous literature [7]. On the other hand, no differences were expected between mothers and fathers regardless of child diagnosis, as reported by other Italian studies using the same measure [5,10]. The second aim was to explore the predictive role of child diagnosis, severity of ASD symptoms, and child internal (implicit) attachment representations on maternal and paternal stress. To this purpose, we used two separate hierarchical regression models for mothers (2a) and fathers (2b) and also tested the interaction effects between child diagnosis and attachment representations. Firstly, we expected to find a positive significant effect of ASD symptom severity [11], but not of child diagnosis, on parenting stress. Second, it is hypothesized that at-risk child attachment representations may negatively affect the experience of parental strain, particularly in mothers and fathers of children with ASD, due to their cumulative adverse contribution to interpersonal relatedness dysfunctions.

## 2. Materials and Methods

### Participants

This study is part of a project investigating attachment and parenting in school-age children with typical and atypical neurodevelopment [35]. Specifically, 56 families of school-age children aged 7 to 13 years were selected and contacted through convenience sampling. Four families refused to participate in the study (2 of ASD, 2 of TD group) and two children with ASD and their parents were excluded since they did not complete the assessment. Thus, the final sample of the current study is composed of 23 children with ASD (IQ > 70), 27 with TD, and their mothers (*n* = 50) and fathers (*n* = 50). Italian native speakers’ children were included in this study. We did not include children with a history of psychiatric disorder in the TD group, which was based on parent reports at the recruitment stage. For the ASD group, the exclusion criteria were severe impairment of cognitive functioning and language using the Wechsler Intelligence Scale for Children (WISC), 4th edition.

In addition, the parent-reported Social Responsiveness Scale (SRS) [36] scores were used to screen for socio-communicative difficulties and the presence of restricted and repetitive interests and behaviors in children without ASD. Children with ASD received a certification according to the Diagnostic Statistical Manual of Mental Disorder 5th Edition [3], which was confirmed using the Autism Diagnostic Observation Schedule (ADOS) [37]. In all groups, we used the Raven Colored Progressive Matrices (CPM) and the WISC subscales (Vocabulary and Similarities) [38] to respectively assess children’s non-verbal and verbal intelligence. Descriptive statistics are described in Table 1 and Table 2.

## 3. Procedure

We reached families of children with ASD through two Italian clinical centers for neurodevelopmental disorders. Data were collected from September 2017 to February 2020 in the Trentino and Campania regions (Italy). In the first stage, participation in the study was proposed by clinical psychologists of the clinical centers to parents of children with certified ASD diagnosis who met the inclusion criteria of the study. Clinical psychologists briefly clarified the aims and methodology of the study. Parents who were interested in participating authorized the research staff to contact them by phone to provide detailed information. Families of children with TD were recruited using snowball sampling. For parents who voluntarily decided to receive more information about the research project, an appointment was scheduled. After signing the informed consent, families who agreed to be part of the study were asked to attend a two-hour interview. The semi-structured interview was administered by a trained clinical psychologist, while parents completed the questionnaire in a separate room. This research was accomplished according to the EU General Data Protection Regulation (GDPR) n. 2016/679. The Ethical Committee of the (masked for blind review) approved the study protocol (IRB, 2017-016).

### 3.1. Measures

#### 3.1.1. Social Responsiveness Scale 2 (SRS)

ASD severity was assessed by using the Social Responsiveness Scale-2 [36], a widely used questionnaire designed to measure restriction of social abilities and repetitive behaviors in children from 4 to 18 years old. It consists of 65 items on a 4-point Likert-type scale (from 1 = “never true”, to 4 = “almost always true”). In this study, we used the SRS total score obtained from five subscales: (1) social awareness; (2) social cognition; (3) social communication; (4) social motivation, and (5) restricted interests and repetitive behavior. Higher scores on this scale indicate greater difficulties in child social responsiveness. Raw scores were converted into T-scores before conducting the analyses. The cut-off of 84.0 was used to screen for ASD in children with typical development. In the current study, mothers completed the Italian version of the questionnaire, which showed Cronbach alpha values of 0.89.

#### 3.1.2. School-Age Assessment of Attachment (SAA)

For the assessment of children’s internal attachment representations, the School-age Assessment of Attachment [39] clinical tool was used. The SAA is a semi-structured interview developed to assess the quality of attachment (self-protective strategies) in children from 6 to 13 years using the Dynamic Maturational Model of Attachment [40]. The SAA consists of a set of seven cards on potential age-salient threatening situations that school-age children may frequently face with the aim to elicit children’s attachment representations (e.g., the child going out alone; being bullied; the father leaving the home; and the mother going to the hospital). Two distinct versions of the cards are used for females and males. The pictures on the card are very simple (black and white) to prevent distractibility and overstimulation. Firstly, the interviewer asks the child to invent a fantasy story based on the card and then to tell a recalled episode on the same topic related to a personal experience. The SAA is audio recorded, transcribed verbatim, and blind coded based on the DMM method derived from the Adult Attachment Interview (DMM-AAI) [41]. The markers were assigned to implicit memory systems (procedural and perceptual/imaged memory), explicit memory systems (semantic and episodic memory), and reflective integration [42].

The primary coder assessed all the transcripts, whereas the secondary coder examined 25% of the total sample. We examined interrater agreement between the two coders by using Cohen’s k coefficient. A continuous variable (SAArisk) which reflects the degree of risk of attachment representations was used as the main outcome in this study. The SAA psychometric properties were confirmed both in clinical and normative groups [39,43].

#### 3.1.3. Parenting Stress Index—Short Form (PSI-SF)

The Parenting Stress Index—Short Form (PSI-SF) [44] is a widely used self-reported questionnaire that consists of 36 items rated on a 5-point Likert-type scale ranging from 1 (strongly agree) to 5 (strongly disagree). The PSI-SF generates three subscales focused on different types and sources of stress that accompany the experience of parenting: (1) Parental Distress (PSI-PD); (2) Parent–Child Dysfunctional Interaction (PSI-P-CDI); and (3) Difficult Child. The sum of the three subscales (i.e., PD, P-CDI, and DC) yields to a PSI-Total scale. This scale has demonstrated adequate psychometric properties in terms of reliability and validity [44]. Moreover, PSI scales have been widely used in previous studies on parents of children with disabilities, including ASD research [2,8]. In this study, parents completed the Italian version of the questionnaire [45], which has shown good internal consistency with alpha values ranging from 0.71 (PD subscale) to 0.89 for mothers and from 0.71 (P-CDI subscale) to 0.89 for fathers.

### 3.2. Data Analysis

Firstly, we examined whether kurtosis and skewness values were within the range of −1 to +1.

Then, differences between ASD and TD groups with respect to the control variables (maternal and paternal age, child age and gender, verbal and non-verbal intelligence, and family economic status) were tested to check for potential confounders. To this purpose, the t-test and chi-squared test were used, respectively, for continuous and categorical variables. Next, bivariate correlational was used to test the associations among the study variables. With respect to the first study aim, we ran a two-way MANOVA to examine group differences in parenting stress total scores and subscales (PD, P-CDI, and DC), testing the main effect of group (ASD and TD), gender (mothers and fathers), and their interaction (gender X group). According to the second aim of the study, two separate hierarchical linear regressions were conducted to assess the effect of specific child predictors on parenting stress (dependent variable) of mothers (2a) and fathers (2b) using the PSI total score. In the first block child diagnosis (ASD vs. TD) was entered, while the second block included ASD symptom severity. In the third block, implicit attachment representations were added to the model. Finally, the interaction between child diagnosis and SAA risk was tested in the fourth block of the regression. Due to the wide age range of the children, we added this variable to the model as a confounding factor. The inclusion of the variables was forced according to our research purposes and statistical analyses were conducted using the SPSS package (25.0 for Windows).

## 4. Results

Descriptive statistics of parents’ and children’s variables were reported, respectively, in Table 1 and Table 2 for both study groups. Regarding group matching, no significant differences emerged between children with ASD and TD based on child age, gender, verbal and non-verbal intelligence, family socioeconomic status, and maternal and paternal age (*p* > 0.05). Preliminary analyses revealed significant differences between groups on SAArisk, with children with ASD showing higher levels of at-risk attachment representations (t_(49)_ = −3.32, *p* = 0.002).

Correlations among the study variables for both study groups are displayed in the Supplementary Materials.

With respect to the first study aim, the two-way MANOVA (Group × Gender) showed statistically significant differences between the two groups in parenting stress (F(3, 92) = 6.66, *p* < 0.001; Wilk’s Λ = 0.77, partial η2 = 022), whereas no effect of parent gender (F(3, 92) = 0.44, *p* < 0.77; Wilk’s Λ = 0.98, partial η2 = 0.019) and no interaction between group and gender (F(3, 92) = 0.94, *p* < 0.44; Wilk’s Λ = 0.96, partial η2 = 0.039) were found. Specifically, univariate analysis showed a main effect of group on Parent–Child Dysfunctional Interaction (F(3, 99) = 20.83, *p* < 0.001, partial η2 = 0.18) and Difficult Child subscales (F(3, 99) = 18.49, *p* < 0.001, partial η2 = 0.16) as well as on the Total Stress scale, (F(3, 99) = 14.48, *p* < 0.001, partial η2 = 0.13), with parents of children with ADS reporting higher scores compared with parents of children with TD. No differences based on group emerged on the Parental Distress subscale (F(3, 99) = 1.18, *p* < 0.20, partial η2 = 0.017).

**Figure 1:**
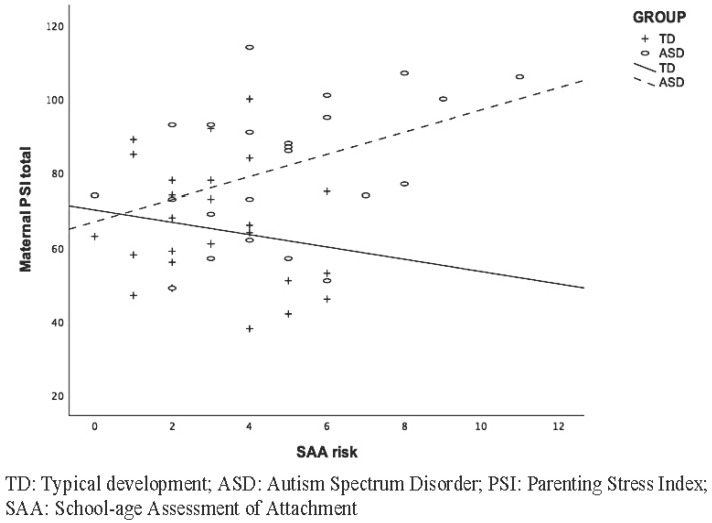
Interaction effect of ASD diagnosis and attachment representation risk (SAA risk) on total parenting stress reported by mothers.

According to the second study aim, we tested the effect of specific child predictors (the presence of ASD diagnosis, ASD symptom severity, and internal attachment representations) on Total Parenting stress using two separate models for mothers and fathers. The hierarchical linear regression on maternal parenting stress (Table 3) showed a statistically significant overall model (F(4, 48) = 6.60, *p* < 0.001), explaining 37% of the variance. In the first step, child diagnosis (β = 0.42, *p* < 0.01, R^2^ = 0.17) significantly predicted parental stress, whereas it was no longer significant in the second block (F(2, 48) = 8.17, *p* < 0.001) when ASD symptom severity (β = 0.44, *p* < 0.001, R^2^ = 0.26) was added to the model. Specifically, higher SRS total scores predicted greater parental stress in mothers. The second block added a significant amount of variance (R^2^ change = 0.086, *p* = 0.025). In the third block, internal attachment representations (β = 0.20, *p* < 0.14) did not contribute significantly to the model. The last block, which tested the interaction effect between child diagnosis and SAA risk, added a significant contribution to the model, revealing a significant predictive effect on maternal stress (β = −1.13, *p* = 0.02, R^2^ change = 0.08). The relationship between child internal attachment representations and parenting stress was significant for the ASD but not for the TD group (Figure 1). Moreover, ASD symptom severity also remained significant in the last block (β = 0.47, *p* = 0.01). The control for child age did not change the significance level of the associations in both maternal and paternal hierarchical regression models.

Next, the overall model on paternal parenting stress is statistically significant (F(4, 47) = 5.01, *p* = 0.002) explaining 31% of the variance (Table 4). The first block includes the presence of child diagnosis (group) and only shows a marginal tendency toward significance (β = 0.46, *p* = 0.073, R^2^ = 0.04). The second block includes ASD symptom severity (β = 0.71 *p* < 0.001) and added a significant amount of variance (R^2^ change = 0.21, *p* < 0.001), which constituted the best model fit for predicting paternal stress scores F(6, 47) = 8.85, *p* = 0.001, R^2^ = 0.28). The third (R^2^ change = 0.01, *p* = 0.46) and fourth blocks (R^2^ change = 0.02, *p* = 0.19) did not contribute significantly to the overall model. Data revealed that the SRS total score remains the only significant child predictor of paternal stress across blocks, maintaining a significant predictive effect in the final model (β = 0.72, *p* = 0.001), with higher ASD symptom severity associated with an increase in paternal stress scores.

## 5. Discussion

Although the study of parenting stress has received much attention in the ASD literature, research is still dominated by maternal stress since the number of fathers is often relatively small and unbalanced [14]. Previous results on gender differences in parenting stress between mothers and fathers of children with ASD [2,11] revealed mixed findings, suggesting the need to understand specific factors associated with parenting stress in both parents. On this matter, it has been found that several child characteristics, besides cognitive abilities and ASD symptom severity, may be associated with parenting stress, playing a different role in mothers and fathers [11,18,19,20,46]. To date, only a few studies have addressed the relationship between child attachment and parenting stress in families of school-age children with ASD and they only considered explicit self-reported attachment measures [21].

### 5.1. Parenting Stress in Mothers and Fathers of Children with ASD

Consistent with well-established findings of the ASD literature [7], parents of children with ASD in our study showed higher levels of parenting stress compared with parents of typically developing children, except for the dimension of Parental Distress. Specifically, the dimension of Parental Distress reflects positive and negative feelings resulting from the parental role, such as the sense of competence, restriction, conflict, support, and depression. Thus, the lack of difference between groups on the PSI-PD subscale may be explained by the fact that this dimension is related to parents’ individual internal psychological dispositions and hence less influenced by child characteristics. In fact, as suggested by previous research [21], child factors (e.g., severity of symptoms) rather than parents’ psychological traits (e.g., personality) could have a strong influence on parenting stress in mothers and fathers of children with ASD. Interestingly, our findings (see Supplementary Materials) showed significant correlations between maternal and paternal stress dimensions (PSI-DC, PSI-P-CDI, and Total Stress) only in the ASD group. These correlations suggest a shared perspective between parents of children with ASD with respect to perceived stress arising from child behavioral and interactive difficulties [47].

Furthermore, in line with our hypothesis, no significant differences were found in parenting stress between mothers and fathers in both study groups. This is consistent with other studies showing similar parenting stress levels in mothers and fathers [2,5,10]. Thus, fathers, just like mothers, experience higher parental burdens and demands associated with rearing a child with ASD. Notably, despite being underrepresented in the literature [14], fathers of children with ASD seem to be negatively affected by their challenging and demanding child-rearing tasks. In addition, fathers are assuming increasingly proactive roles, not only in domestic childcare responsibilities but also in engaging with therapeutic intervention within the context of neurodevelopmental disorders [14,48].

Thus, considering their critical influence on child development and family functioning [49], fathers should be regularly engaged in early interventions and receive adequate psychological support to address difficulties associated with raising a child with ASD [50].

### 5.2. Relations between Child Attachment Representations and Parental Stress of Mothers and Fathers

To the best of our knowledge, this is the first study to explore the association between child attachment and parenting stress in ASD, considering internal attachment representations. Firstly, at a preliminary level, children with ASD showed higher at-risk attachment internal representations compared with the control group [35]. This finding could be ascribed to multiple aspects often associated with ASD diagnosis, such as impairments in mentalizing and social information processing [33,51].

With respect to the predictors of parenting stress, ASD symptom severity was found to be associated with higher parenting stress in mothers and fathers. This is consistent with previous findings [2] documenting that child difficulties in social functioning constitute the most consistent predictor of stress in both parents. Thus, problems in social communication and interpersonal relatedness as well as the display of repetitive and restricted interests and behaviors perceived by the mothers seem to increase the overall level of stress related to the parenting role. Interestingly, ASD severity appears to have a greater impact on maternal and paternal stress than the diagnosis itself. In this regard, it becomes essential to consider the degree of child impairment in terms of social reciprocity, awareness, and motivations as well as restricted interests and behaviors to understand the experience of parental burden in both parents.

A pivotal novelty of this study is the application of a semi-structured interview to assess implicit attachment representations in children with ASD. In the current study, at-risk internal attachment representations are associated with higher parenting stress in mothers of children with ASD. This finding contrasts with previous results by Keenan and colleagues [21] who reported no associations between self-reported attachment scores of children with ASD and parenting stress of their primary caregivers (including only one father). In this case, attachment to the primary caregiver was measured in 24 children with high-functioning ASD (aged from 7 to 14) and 24 with typical development by using a self-reported questionnaire. Children reported their perceptions of security in their relationship to an identified attachment figure (explicit attachment representations). No associations were found between child attachment and parenting stress either in TD or in the ASD group. Notably, it is essential to distinguish between perceived attachment as reported by the child through standardized questionnaires (explicit representations) and internal attachment representation, which are mainly automated and unconscious, consisting of a processing system model of self, others, and the context. On this matter, attachment self-report measures may not be sensitive in capturing school-age children’s internal attachment representations, which may be difficult to access on a conscious level [31].

According to our results, prior research revealed that at-risk attachment is associated with higher parenting stress in typical development [23] and may also have a detrimental effect on parenting in the context of atypical development. Different aspects that may characterize more insecure implicit attachment representations can constitute a significant source of stress for mothers in the context of ASD such as difficulties in emotion regulation, hypo or hyperarousal activation, poor self-knowledge, and mentalizing [33]. In addition, at-risk implicit attachment representations expose parents of children with ASD to additional challenges making it more difficult to interpret and respond to children’s needs for protection and comfort, therefore increasing the risk of relational disruptions (omitted for blind review).

For instance, children’s rigid interpersonal expectations and difficulties in the interpretation of the social environment may lead to more frequent disruptions and less sustained positive engagement in the parent–child relationship [52]. Moreover, discriminating attachment and autism difficulties in children can be very complex [25], increasing the risk of negative feelings, miscommunications, and parent–child mismatches. These unique challenges experienced by parents of children with ASD can make it more complex to understand children’s needs, leading to higher strain and feelings of incompetence [53].

Taken together, these distinctive characteristics may explain the way in which at-risk internal attachment representations contribute to stressful and demanding parenting experiences. Since we did not find a similar effect in parents of children with TD, difficulties of children with ASD in interpreting parental goals and intentions as well as in signaling their affective needs may also explain the higher level of at-risk attachment representations and in turn of parenting strain.

Nevertheless, no associations between child attachment representations and the parenting stress of fathers were found. It is plausible that other non-ASD score factors instead of attachment can contribute to increased parenting stress in fathers of children with ASD, such as externalizing behaviors [2,54].

Some limitations of the study should be acknowledged, such as the wide age range of the children, the small sample size, and the lack of a measure assessing parenting style and caregiving roles. The cross-sectional design did not allow us to consider the predictive effects across time, only highlighting associations among the study variables. In addition, we did not consider the potential confounding role of comorbidity in children with a diagnosis of ASD.

## 6. Conclusions

The current study contributed to the field of family processes in the context of ASD by examining the influence of child attachment representations on parenting stress and by including both mothers and fathers, using a balanced sample. According to previous studies, parents of children with ASD reported higher stress compared with parents of children with TD. No differences were found between mothers and fathers, suggesting that rearing a school-age child with ASD (without intellectual disability) may constitute a relevant source of stress regardless of parent gender. Implicit attachment representations are associated with parenting stress only in mothers. This suggests the potential benefit of adaptive attachment representations not only for children themselves but also for mothers and the family system since parenting stress levels have been found to be correlated among parents. For this reason, child interpersonal expectations, regulatory strategies, and self-protective organization should be considered in the psychological assessment with the aim of tailoring personalized interventions for families of children with ASD. Moreover, these results confirm the need to use a multimodal assessment to capture different components and processes related to attachment, providing encouraging results concerning the use of the SAA in the context of ASD. Future studies should replicate and extend these findings, using larger sample sizes, longitudinal designs, and sophisticated statistical models to address bidirectional effects between parent and child dimensions. In addition, further research on ASD should consider additional factors that could make the experience of parenting more stressful (e.g., paternal involvement) by examining the influence of parental stress on maternal and paternal responsiveness, attunement, and sensitivity toward their child.

Taken together, our findings suggest that ecological approaches to determinants of parenting stress in families of children with ASD should include specific factors explaining individual psychological functioning, such as child attachment representations. Finally, the different impact of child variables on parenting stress confirms the need to assess the uniqueness of maternal and paternal parenting experiences in the context of ASD, both in clinical and research practice.

## Figures and Tables

**Table 1:** Descriptive statistics of maternal and paternal variables for both study groups.

	Mothers				Fathers			
	ASD (n = 23)		TD (n = 27)		ASD (n = 23)		TD (n = 27)	
	*M*	*SD*	*M*	*SD*	*M*	*SD*	*M*	*SD*
Age	43.0	5.72	44.12	4.35	46.23	7.07	47.45	5.89
PSI-Total Stress	81.61	18.65	65.15	16.26	74.22	15.36	64.96	16.69
PSI- Parental Distress	25.35	8.38	23.30	6.56	23.65	6.58	22.04	7.14
PSI- Parent-Child Dysfunctional Interaction	25.30	6.76	18.93	5.24	23.09	4.79	19.38	5.02
PSI- Difficult Child	30.96	7.98	22.59	6.59	27.48	6.97	23.54	6.88

ASD: Autism Spectrum Disorder; TD; Typical Development; PSI: Parenting Stress Index.

**Table 2:** Descriptive statistics of the child variables for both study groups.

	ASD (n = 23)		TD (n = 27)		Group comparison	
	*M*	*SD*	*M*	*SD*	*t*	*p*
Age (months)	124.46	17.38	118.44	24.06	−1.01	0.31
Family SES	42.02	14.54	44.66	14.80	0.64	0.52
Nonverbal IQ	0.41	0.86	0.59	0.88	0.70	0.48
Verbal IQ	22.45	4.00	22.48	4.67	0.02	0.98
SRS	76.52	17.14	47.30	7.93	−7.92	<0.001
SAA risk	5.00	2.58	3.00	1.66	−3.32	0.002 **

ASD: Autism Spectrum Disorder; TD; Typical Development; SRS: Social Responsiveness Scale; SAA: School-age Assessment of Attachment; ** *p* <0.01.

**Table 3:** Hierarchical linear regression predicting parenting stress in mothers.

	Total Parenting Stress							
	Model 1		Model 2		Model 3		Model 4	
*Variables*	β	*CI*	β	*CI*	β	*CI*	β	*CI*
Child diagnosis	0.42 **	2.91; 13.02	0.08	−5.76; 8.95	−0.01	−7.75; 7.52	−0.55	−21.89; 0.99
ASD severity			0.44 *	0.05; 0.80	0.44 *	0.06; 0.80	0.47 *	0.10; 0.80
SAA risk					0.20	−0.61; 4.00	−0.55	−10.35; 1.21
SAA risk X Child diagnosis							1.13 *	0.39; 5.03
*ΔR^2^*	0.176 *		0.086 **		0.034		0.079 *	
*R^2^*	0.176		0.262		0.296		0.375	

ASD: Autism Spectrum Disorder; SAA: School-age Assessment of Attachment. * *p* < 0.05, ** *p* <0.01.

**Table 4:** Hierarchical linear regression predicting parenting stress in fathers.

	Total Parenting Stress							
	Model 1		Model 2		Model 3		Model 4	
*Variables*	β	*CI*	β	*CI*	β	*CI*	β	*CI*
Child diagnosis	0.26	−0.42; 8.95	−0.27	−10.96; 1.82	−0.32	−12.00; 1.44	−1.75	−21.07; 0.01
ASD severity			0.71 ***	0.26; 0.91	0.71	0.26; 0.91	0.69	0.27; 0.92
SAA risk					0.10	−1.27; 2.74	−0.40	−7.68; 2.85
SAA risk X Child diagnosis							0.81	−0.74; 3.46
*ΔR^2^*			0.214 ***		0.009		0.027	
*R^2^*	0.068		0.282		0.291		0.318	

ASD: Autism Spectrum Disorder; SAA: School-age Assessment of Attachment. *** *p* < 0.001

## Supplementary Materials

The following supporting information can be downloaded at: https://www.mdpi.com/article/10.3390/children10101633/s1, Table S1: Bivariate correlations for the autism spectrum disorder (ASD) group; Table S2: Spearman correlations for the control group.
