# Supplementary material for: Child Attachment Representations and Parenting Stress in Mothers and Fathers of School-Age Children with a Diagnosis of Autism Spectrum Disorder: A Pilot Cross-Sectional Study
Source: Children (Basel). 2023 Sep 30;10(10):1633. doi: 10.3390/children10101633 (PMC10605204; doi:10.3390/children10101633)
Supplement: Supplementary file 1 [file children-10-01633-s001.zip › children-2579543-supplementary.pdf]

Table S1. Bivariate correlations for the autism spectrum disorder (ASD) group

	SRS	SAA risk	PSI Total mother	PSI_PD mother	PSI_P-CDI mother	PSI_DC mother
SRS	-		0.323	0.101	0.156	0.526*
			0.143	0.655	0.487	0.012
SAA risk	-0.057	-	0.413	0.294	0.275	0.424*
	0.795		0.050	0.173	0.203	0.044
PSI Total father	0.580**	0.182	0.425*	0.208	-0.153	0.645**
	0.005	0.406	0.043	0.341	0.487	0.001
PSI_PD father	0.432*	0.207	0.310.	0.352	-0.052	0.399
	0.045	0.343	0.350	0.099	0.815	0.059
PSI_P-CDI father	0.523**	0.197	0.354	0.055	0.262	0.549**
	0.012	0.368	0.097	0.804	0.228	0.007
PSI_DC father	0.503**	0.070	0.399	0.088	0.202	0.667**
	0.017	0.752	0.059	0.690	0.347	0.001

SRS: Social Responsiveness Scale; SAA: School-age Assessment of Attachment; PSI: Parenting Stress Index; PSI_PD: Parental Distress subscale; PSI_P-CDI: Parent-Child Dysfunctional Interaction subscale; PSI_DC: Difficult Child subscale.

*p < .05, ** p <.01, ***p < .001

Table S2. Spearman correlations for the control group

	SRS	SAA risk	PSI Total mother	PSI_PD mother	PSI_P-CDI mother	PSI_DC mother
SRS	-		0.368	0.368	0.113	0.468
			0.059	0.059	0.576	0.014
SAA risk	-0.087	-	0.170	-0.250	0.128	0.424*
	0.665		0.395	0.209	0.525	0.044
PSI Total father	0.433*	0.070	-0.018	0.050	-0.174	0.021
	0.027	0.735	0.931	0.810	0.395	0.921
PSI_PD father	0.273	0.199	-0.010	0.124	-0.237	-0.010
	0.177	0.329	0.960	0.545	0.244	0.963
PSI_P-CDI father	0.435*	0.036	-0.220	0.185	0.247	-0.128
	0.027	0.861	0.279	0.366	0.224	0.532
PSI_DC father	0.449*	0.012	0.129	0.126	0.003	0.153
	0.021	0.954	0.532	0.540	0.988	0.454

SRS: Social Responsiveness Scale; SAA: School-age Assessment of Attachment; PSI: Parenting Stress Index; PSI_PD: Parental Distress subscale; PSI_P-CDI: Parent-Child Dysfunctional Interaction subscale; PSI_DC: Difficult Child subscale.
*p < .05, ** p <.01, ***p < .001
